# Transcriptomic analysis of *Vigna radiata* in response to chilling stress and uniconazole application

**DOI:** 10.1186/s12864-022-08443-6

**Published:** 2022-03-14

**Authors:** Hanqiao Hu, Naijie Feng, Xuefeng Shen, Liming Zhao, Dianfeng Zheng

**Affiliations:** 1 Department of Biotechnology, College of Coastal Agricultural Sciences, Guangdong Ocean University, Guangdong 524088 Zhanjiang, China; 2 Shenzhen Research Institute of Guangdong Ocean University, Shenzhen, 518108 China

**Keywords:** *Vigna radiata*, Uniconazole, Transcriptome, Yield, Chilling stress

## Abstract

**Background:**

Chilling injury of mung bean (*Vigna radiata* (L.)) during the blooming and podding stages is a major agricultural threat in Northeast China. Uniconazole (UNZ) can alleviate water deficit stress in soybean and waterlogging stress in mung bean. However, there has been no report on the effect of UNZ application on the growth and transcriptomic profile of mung bean under chilling stress.

**Results:**

UNZ application before chilling stress at the R1 stage alleviated the decline in mung bean yield. UNZ delayed the decrease in leaf chlorophyll content under chilling stress at the R1 stage and accelerated the increase in leaf chlorophyll content during the recovery period. Eighteen separate RNA-Seq libraries were generated from RNA samples collected from leaves exposed to six different treatment schemes. The numbers of DEGs specific for UNZ treatment between D1 + S vs. D1 and D4 + S vs. D4 were 708 and 810, respectively. GO annotations showed that photosynthesis genes were obviously enriched among the genes affected by chilling stress and UNZ application. KEGG pathway enrichment analysis indicated that 4 pathways (cutin, suberin and wax biosynthesis; photosynthesis; porphyrin and chlorophyll metabolism; and ribosome) were downregulated, while plant–pathogen interaction was upregulated, by chilling stress. UNZ application effectively prevented the further downregulation of the gene expression of members of these 4 KEGG pathways under chilling stress.

**Conclusions:**

UNZ application effectively delayed the decrease in photosynthetic pigment content under chilling stress and accelerated the increase in photosynthetic pigment content during the recovery period, thus effectively limiting the decline in mung bean yield. UNZ application effectively prevented the further downregulation of the gene expression of members of 4 KEGG pathways under chilling stress and increased mung bean tolerance to chilling stress.

**Supplementary Information:**

The online version contains supplementary material available at 10.1186/s12864-022-08443-6.

## Background

Mung bean (*Vigna radiata* L.) is an important and valuable legume crop due to its nutritional and health benefits. Mung beans contain significant amounts of proteins and phytochemicals with beneficial activities [1]. According to the Food and Agriculture Organization of the United Nations, in 2019, the global planting area of mung bean was 1649.7 thousand hectares, and the output was 26.982 million tons. Mung bean is one of the most important legume crops in China and is mainly produced in the Huang Huai River Basin and in Northeast China [2]. Mung bean originated in the tropics and is a thermophilic crop.

Cold stress can be classified as chilling (0–15 °C) or freezing (< 0 °C) stress, and both of these can cause significant crop loss. Chilling injury is a major agricultural hazard in the summer in Northeast China [3]. During the period from soybean sowing to maturity, periodic low-temperature chilling injury often occurs, mainly in the blooming and podding stages, which often reduces yield and quality [4–7]. Cold stress responses are influenced by the exposure duration [8], plant species [9] and plant development stage [7]. The exposure of mung bean to chilling results in high and irreversible electrolyte leakage [10]. The susceptibility of mung bean to chilling stress is related to the differential expression of cold-related genes [11, 12].

It has been well documented that plant growth regulators (PGRs) play important roles in crop production and in resistance to environmental stresses. Uniconazole (S-3307, UNZ), a plant growth retardant, has been increasingly applied in crops to increase their output and quality [13–15]. In a previous study, soybean plants were treated with UNZ at 50 mg L^−1^ at the beginning of bloom and then exposed to water deficit stress for 7 d. The chlorophyll content and photosynthesis rate were decreased by water deficit stress but remained higher in UNZ-treated stressed plants than in the stressed control. Biomass accumulation and seed yield were also increased in plants treated with UNZ compared with controls [15]. Mung bean plants were treated with UNZ at 50 mg L^−1^ at the beginning of bloom and at the beginning of seed development and then exposed to waterlogging for 5 days. The chlorophyll content and photosynthetic rate were decreased by waterlogging stress but remained higher in UNZ-treated plants than in the stressed control. Thus, UNZ could effectively alleviate yield reduction [16].

The genome of mung bean has been recently sequenced [17], which is a valuable resource for research on stress tolerance genes in plants. RNA sequencing (RNA-seq) analysis is a powerful method for transcriptome analysis that has been increasingly used to study transcriptomic responses to chilling/cold stress in many legume species [15, 18–21]. Transcriptome analysis has been conducted to analyze desiccation tolerance, adventitious rooting and bruchid resistance in mung bean [22–24]; however, the effect of UNZ on the growth and the transcriptomic profile of mung beans under chilling stress has not yet been investigated. In this work, mung bean plants were predicted to be harmed by chilling stress at the blooming and podding stages, and the application of UNZ could alleviate stress damage in soybean. The yield, photosynthetic pigment content and relevant transcriptome changes in mung bean in response to UNZ under low-temperature stress were studied. Thus, this study aimed to identify differentially expressed genes (DEGs) that influenced yield and photosynthetic pigments via transcriptome analysis, which would be useful for the application of UNZ in the production of mung bean. At the same time, it provides some reference information for mung bean cultivation in Heilongjiang and other cold regions.

## Results

### Effects of chilling stress and UNZ application on yield and yield components in mung bean seedlings

Pod number per plant and particle number per plant are two important yield components. The effect of UNZ on yield and yield components in mung bean under chilling stress at R1 stage in 2017 is shown in Table 1. The yields of the two mung bean varieties were significantly lower than those of CK after chilling treatment at stage R1. The pod number per plant, particle number per plant and yield followed the same pattern in the 2 varieties, specifically, CK > D1 > D2 > D3 > D4. The yield loss of Lvfeng 2 was more severe, which indicated that the different genotypes of mung bean had different tolerances to chilling stress. The yield loss in 2017 was similar to that in 2016 (data not shown). However, spraying UNZ at the R1 stage effectively alleviated the decline in mung bean yield.

**Table 1:** Effect of chilling stress and UNZ application on yield and yield components in mung bean at the R1 stage in 2017

Varieties	Treatments	Pod number per plant (PCS/plant)	Particle number per plant (PCS/plant)	Yield (g/plant)
Lvfeng 2	CK	17.59 ± 0.30a	132.11 ± 8.45a	5.39 ± 0.10a
	D1	17.10 ± 0.25a	113.50 ± 6.78b	4.90 ± 0.09b
	D1 + S	18.40 ± 0.19a	129.80 ± 8.23a	5.38 ± 0.11a
	D2	15.80 ± 0.15b	105.90 ± 9.34c	4.19 ± 0.13d
	D2 + S	18.14 ± 0.10a	119.86 ± 10.03b	4.86 ± 0.08b
	D3	14.50 ± 0.22b	102.50 ± 9.54c	4.15 ± 0.05d
	D3 + S	14.90 ± 0.16b	122.80 ± 10.11ab	4.79 ± 0.05b
	D4	9.80 ± 0.09c	67.70 ± 5.34d	2.97 ± 0.03e
	D4 + S	13.50 ± 0.11bc	103.90 ± 9.63c	4.31 ± 0.02 cd
Lvfeng 5	CK	20.38 ± 0.26a	112.63 ± 10.10a	5.34 ± 0.10a
	D1	10.71 ± 0.12b	95.00 ± 7.34b	4.85 ± 0.08ab
	D1 + S	10.67 ± 0.08b	70.83 ± 4.97c	5.16 ± 0.11a
	D2	8.76 ± 0.04bc	56.00 ± 6.87de	4.72 ± 0.08ab
	D2 + S	9.25 ± 0.09b	61.50 ± 5.55d	5.02 ± 0.10a
	D3	8.67 ± 0.12bc	58.00 ± 4.98de	4.22 ± 0.07b
	D3 + S	8.38 ± 0.11bc	72.25 ± 8.36c	4.47 ± 0.08b
	D4	7.11 ± 0.07c	54.67 ± 6.38e	3.40 ± 0.09c
	D4 + S	6.66 ± 0.05c	44.17 ± 7.70f	4.33 ± 0.06c

*Note:* The data represent the mean ± SD of four replicates. CK, plant growth in the natural environment; D, plant growth in the chilling environment; D + S, plant growth in the chilling environment + 50 mg·L ^−1^ UNZ; 1–4, time for treatment. Values within the same column followed by different letters are significantly different at the 0.05 level

### Effects of chilling stress and UNZ application on photosynthetic pigments in mung bean at the R1 stage

**Fig. 1:**
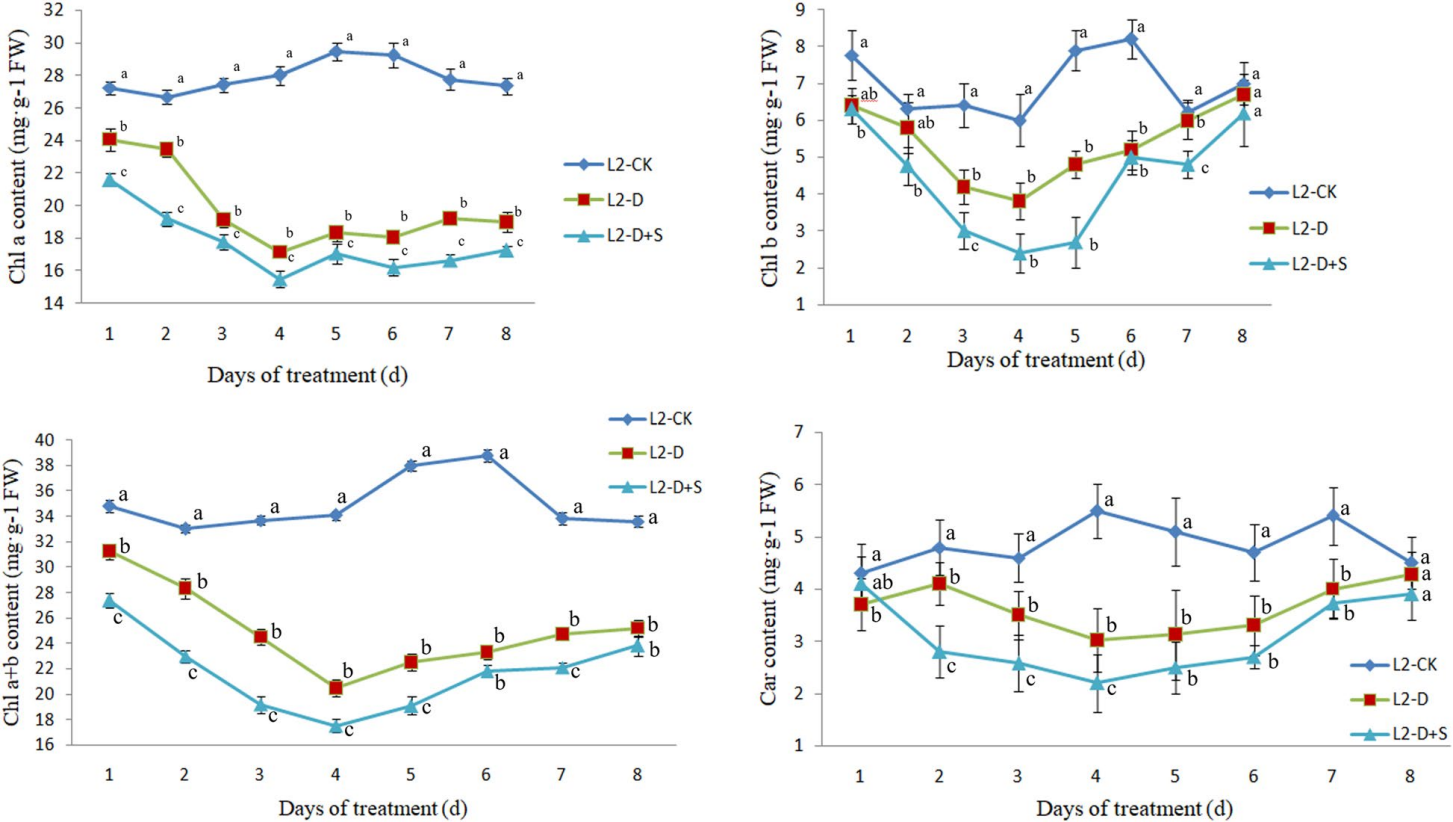
Effects of uniconazole on photosynthetic pigments in Lvfeng 2 under chilling stress at the R1 stage. Note: Data in the figure are the mean ± SD of four replicates. L2-CK, plants grown in the natural environment; L2-D, plants grown in the chilling environment; L2-D + S, plants grown in the chilling environment + 50 mg·L-1 UNZ; 1–4, time for chilling stress; 5–8: time for recovery in the natural environment. Different lowercase letters represent significant differences (p < 0.05) between the treatment and control

Chlorophylls and carotenoids are the main photosynthetic pigments in plants. Photosynthetic pigments are involved in the process of absorbing and transferring energy. The change in photosynthetic pigment content can reflect the degree of chilling stress. The contents of chlorophyll a (Chl a) and total chlorophyll (Chl (a + b)) of Lvfeng 2 (L2) in L2-D and L2-D + S were significantly lower than those in the control from 1 to 8 d of treatment. However, the contents of chlorophyll b (Chl b) in L2-D and L2-D + S were significantly lower than those in the control from 3 to 7 d of treatment. The contents of total carotenoids (Car) in L2-D and L2-D + S were significantly lower than those in the control from 2 to 7 d treatment. The contents of Chl b and Car reached the normal level, that is, equivalent to the control, after 4 d of recovery. The contents of Chl a and Chl (a + b) in L2-D were significantly lower than those in L2-D + S from 1 to 8 d of treatment. However, the contents of Chl b in L2-D were significantly lower than those in L2-D + S from 3 to 7 d of treatment, and the contents of total Car in L2-D were significantly lower than those in L2-D + S from 2 to 4 d of treatment. Generally, the contents of Chl a, Chl b, Chl (a + b) and total Car decreased gradually with the extension of chilling stress and increased with the extension of recovery time but were always lower than those in the control (Fig. 1). The changes in the contents of Chl a, Chl b, total Chl and Car in Lvfeng 5 (Fig. S1) in L2-D and L2-D + S were similar to those in Lvfeng 2. Exogenous application of UNZ can effectively delay the decreases in the photosynthetic pigment content of leaves under chilling stress at the R1 stage and accelerate the increase in photosynthetic pigment content in leaves during the recovery period.

### Transcriptome profiles of mung bean leaves under chilling stress and UNZ application

An overview of the RNA-seq reads derived from the eighteen libraries is presented in Table 2. A total of 133.08 Gb of clean reads was obtained, with an average of 22.18 Gb of reads for each sample. The average Q30 was 94.28%, and the GC content of each sample was uniform and stable, which indicated that the sequencing results were highly accurate.

**Table 2:** Summary of raw RNA-seq reads of mung bean leaves under chilling stress and UNZ application

Sample	Raw Reads	Clean Reads	Clean Bases (G)	Q30 (%)	GC Content	Mapped Ratio (%)	Unique Mapped Ratio (%)
CK1	24,527,414.33	23,928,610.67	7.18	93.48	45.04	92.80	88.48
CK4	21,488,160.33	21,026,071.33	6.31	93.87	46.17	93.28	88.13
D1	23,158,506.00	22,613,379.00	6.79	94.72	44.76	93.27	90.50
D4	22,417,583.33	21,807,628.00	6.54	94.10	44.42	92.22	87.24
D1 + S	22,146,488.33	21,571,532.33	6.47	94.87	44.53	92.91	88.29
D4 + S	22,722,631.33	22,132,827.33	6.64	94.62	44.53	93.18	88.81

*Note:* GC content: Clean Data G and C percentage of the total bases; Q30: Quality Score of base is greater than or equal to 30% of the total bases

The clean reads of each library were mapped to the reference genome sequence of mung bean. The mapped ratio of the libraries ranged from 92.22% to 93.28%, and the uniquely mapped ratio of the libraries ranged from 87.24% to 90.50%. The data were qualified for subsequent analysis. The correlation coefficient of the three biological repeats in each treatment was more than 0.9 (Fig. S2).

The numbers of DEGs between different pairs of treatments with FDR < 0.01 and fold change ≥ 2 are shown in Table S2. The number of DEGs between D1 + S and CK1 was 4 905, while the number of DEGs between D1 and CK1 was 4 025. The number of DEGs decreased to 3 266 in D4 + S vs. CK4 compared to the 4 023 DEGs in D4 vs. CK4.

**Fig. 2:**
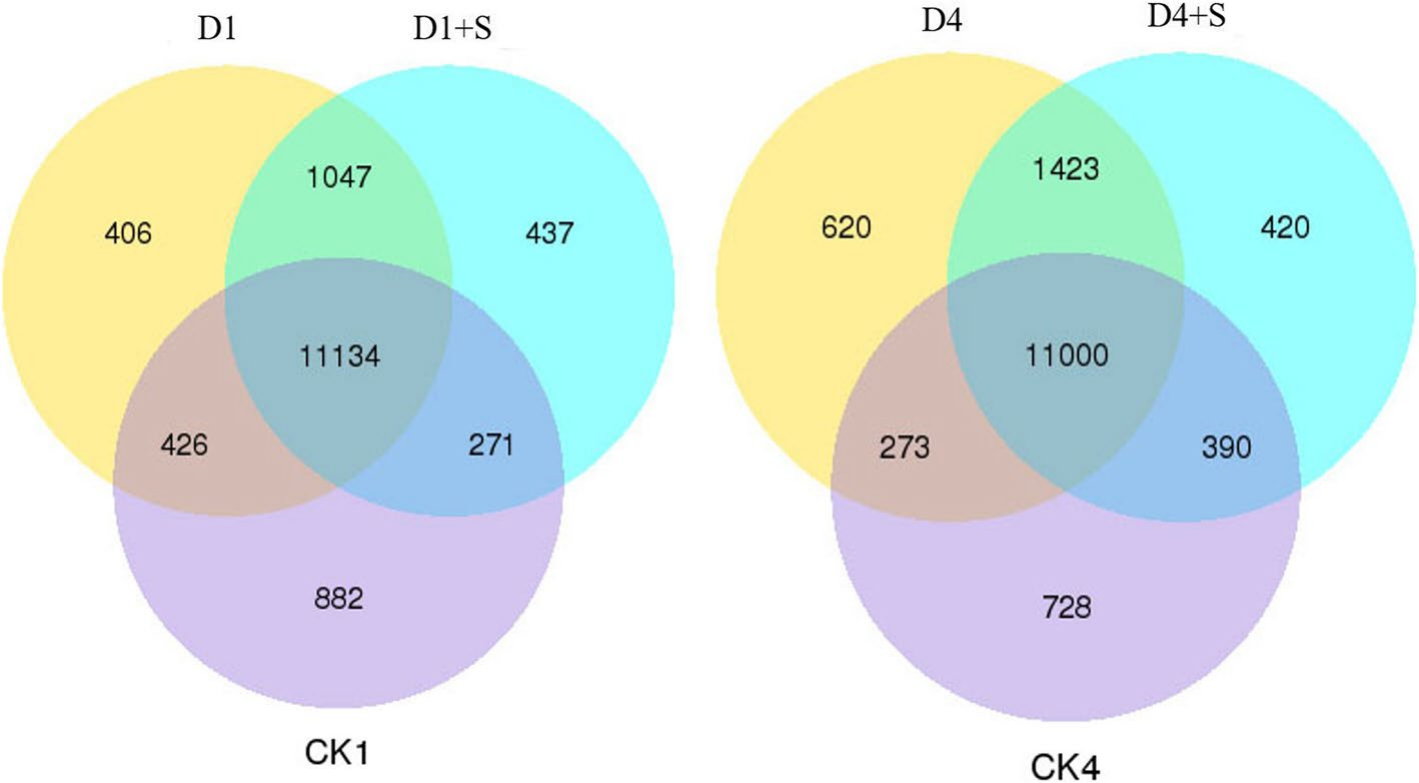
Venn diagram of the differentially expressed genes of mung bean leaves under chilling stress and UNZ application. Note: CK, plants grown in the natural environment; D, plants grown in the chilling environment; D + S, plants grown in the chilling environment + 50 mg·L-1 UNZ; 1 and 4, 1 d and 4 d of chilling stress

The numbers of shared DEGs in mung bean leaves among these treatments were 11,134 and 11,000 for 1 day of chilling and 4 days of chilling stress, respectively. which may reflect that the expression of these genes was not influenced by UNZ or chilling stress. The numbers of DEGs specific to UNZ treatment between D1 + S and D1 and between D4 + S and D4 were 708 and 810, respectively (Fig. 2).

### GO enrichment analysis of DEGs in mung bean leaves under chilling stress and UNZ application at the R1 stage

GO annotations were used to classify the possible functions of the mung bean genes. GO functional enrichment indicated that 1170, 1184, 2017, and 1385 DEGs in D1-CK1, D1 + S-CK1, D4-Ck4 and D4 + S-CK4, respectively, were classified into the three GO categories of biological process (BP), cellular component (CC), and molecular function (MF). The significantly enriched GO categories in CC, BP and MF are shown in Table 3. Only GO terms in the MF category were significantly enriched in D1 vs. CK1 and D1 + S vs. CK1 among the three categories. The number of enriched GO terms in the MF category was greater in D1 + S vs. CK1 than in D1 vs. CK1, which implies that the application of UNZ plays an important role in regulating MF gene expression under chilling stress. In D4 vs. CK4 and D4 + S vs. CK4, GO terms in each of the three categories were significantly enriched, and the number of GO terms in the CC category was the highest among the 3 categories. It is obvious that the cell components related to photosynthesis were most influenced by chilling stress.

**Table 3:** The significantly enriched GO pathways

Combination	category	GO ID	Description	Q value
D1 vs. CK1	MF	GO:0,016,747	transferase activity, transferring acyl groups other than amino-acyl groups	0.007
D4 vs. CK4	BP	GO:0,015,979	photosynthesis	5.24E-09
	CC	GO:0,009,521	photosystem	8.86E-09
		GO:0,034,357	photosynthetic membrane	8.86E-09
		GO:0,009,579	thylakoid	1.92E-08
		GO:0,044,436	thylakoid part	1.92E-08
		GO:0,009,522	photosystem I	1.09E-06
		GO:0,009,523	photosystem II	0.000
		GO:0,098,796	membrane protein complex	0.002
		GO:0,009,654	photosystem II oxygen evolving complex	0.002
		GO:0,042,651	thylakoid membrane	0.002
		GO:1,990,204	oxidoreductase complex	0.003
		GO:0,019,898	extrinsic component of membrane	0.030
	MF	GO:0,005,509	calcium ion binding	0.008
D1 + S vs. CK1	MF	GO:0,005,506	iron binding	0.007
		GO:0,004,866	endopeptidase inhibitor activity	0.007
		GO:0,030,414	peptidase inhibitor activity	0.007
		GO:0,061,134	peptidase regulator activity	0.007
		GO:0,061,135	endopeptidase regulator activity	0.007
		GO:0,016,705	oxidoreductase activity, acting on paired donors, with incorporation or reduction of molecular oxygen	0.009
		GO:0,005,509	calcium ion binding	0.0192
		GO:0,009,055	electron carrier activity	0.0192
D4 + S vs. CK4	BP	GO:0,015,979	photosynthesis	1.07E-09
		GO:0,019,684	photosynthesis, light reaction	0.008
		GO:1,901,566	organonitrogen compound biosynthetic process	0.022
	CC	GO:0,034,357	photosynthetic membrane	3.12E-09
		GO:0,009,579	thylakoid	4.64E-09
		GO:0,044,436	thylakoid part	4.64E-09
		GO:0,009,521	photosystem	5.60E-09
		GO:0,009,523	photosystem II	0.000
		GO:0,042,651	thylakoid membrane	0.001
		GO:0,009,522	photosystem I	0.002
		GO:0,005,840	ribosome	0.003
		GO:0,009,654	photosystem II oxygen evolving complex	0.006
		GO:1,990,204	oxidoreductase complex	0.010
		GO:0,030,529	intracellular ribonucleoprotein complex	0.022
		GO:1,990,904	ribonucleoprotein complex	0.022
		GO:0,098,796	membrane protein complex	0.045
	MF	GO:0,005,509	calcium ion binding	3.75E-05
		GO:0,003,735	structural constituent of ribosome	0.008
		GO:0,005,198	structural molecule activity	0.016

### KEGG enrichment analysis of DEGs in mung bean leaves under chilling stress and UNZ application at the R1 stage

**Fig. 3:**
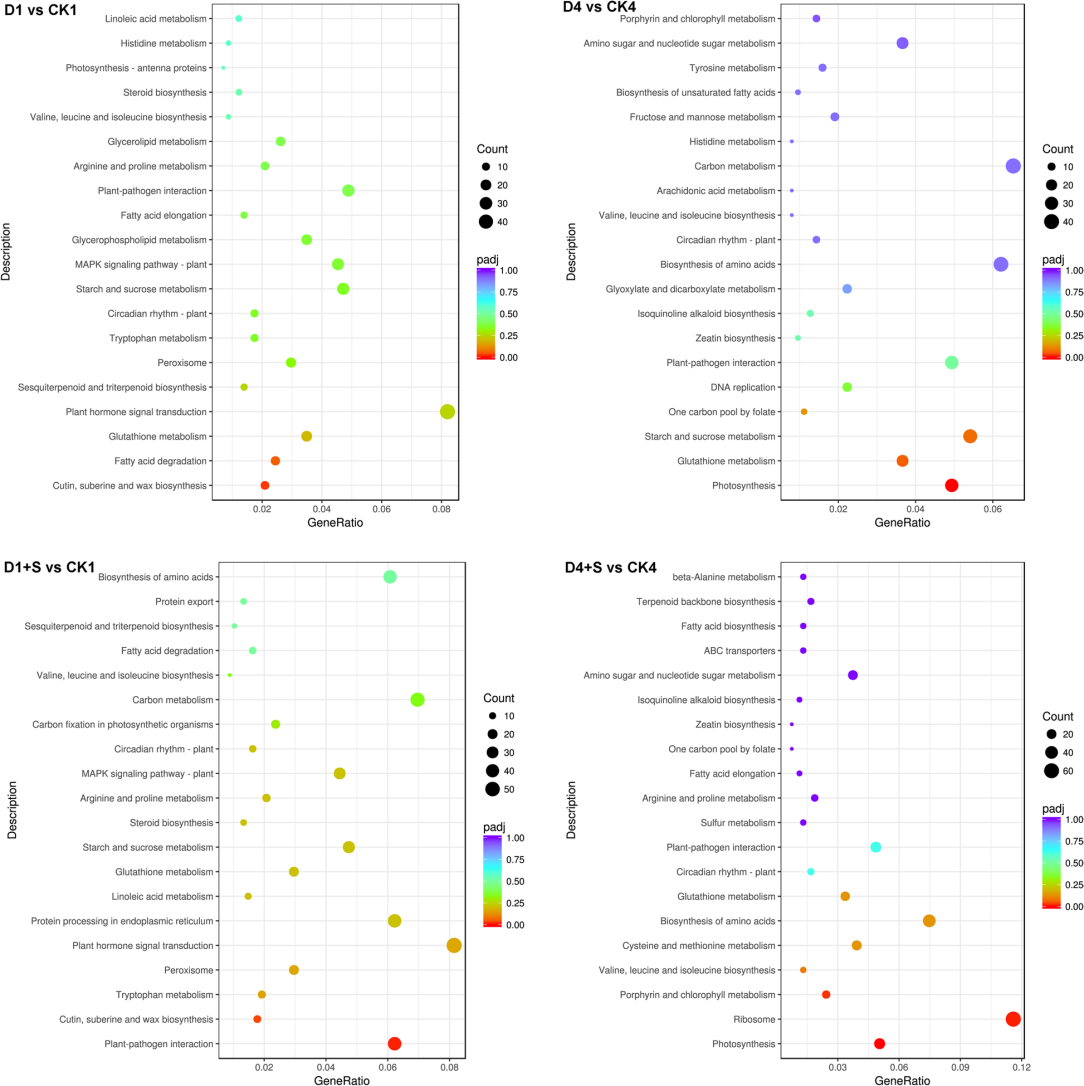
Functional analysis of DEGs based on KEGG pathway annotations. Pathways with a Q-value ≤ 0.05 that were significantly enriched in DEGs in the comparison between the treatment and control at the flowering stages were analyzed with the KEGG database. The degrees of KEGG enrichment can be measured by the gene ratio, padj (multiple testing correction-adjusted p values), and number of genes enriched in this pathway. The right y-axis represents the KEGG pathway, and the x-axis shows the gene ratio, which denotes the ratio of the number of DEGs to the number of annotated genes enriched in this pathway

For the KEGG pathway enrichment analysis [25], pathways with a Q value ≤ 0.05 were regarded as significantly changed in response to chilling stress with or without UNZ treatment. The top 20 enriched KEGG pathways are presented in Fig. 3. The pathways with significant enrichment among the DEGs were cutin, suberin and wax biosynthesis (Vra00073) in D1 vs. CK1; photosynthesis (Vra00195) in D4 vs. CK4; cutin, suberin and wax biosynthesis (Vra00073) and plant vs. pathogen interaction (Vra04626) in D1 + S vs. CK1; and photosynthesis (Vra00195), ribosome (Vra03010) and porphyrin and chlorophyll metabolism (Vra00860) in D4 + S vs. CK4.

### Differential expression of plant–pathogen interaction pathway genes

The expression levels of all plant–pathogen interaction genes except calcium-dependent protein kinase SK5 were upregulated after chilling stress with or without UNZ in all four treatments. These genes are important Ca^2+^ signaling genes, including 7 calcium-binding protein genes, 3 calcium-dependent protein kinase genes and 5 calmodulin calmodulin-like genes.

Two heat shock proteins, heat shock cognate protein and endoplasmin, were upregulated. One mitogen-activated protein kinase, pathogenesis-related gene transcriptional activator, respiratory burst oxidase homolog protein, somatic embryogenesis receptor kinase and WRKY transcription factor each were also upregulated.

The log_2_(fold change) (log_2_(FC)) values of most calmodulin and calmodulin-like proteins were positive in D1 + S vs. D1 and D1 + S vs. D1. In particular, calmodulin in D1 + S vs. D1 and calmodulin-like protein 11 in D1 + S vs. D1 and D4 + S vs. D4 were notably upregulated (log_2_(FC) > 1), indicating that UNZ treatment before chilling stress upregulated the expression of some Ca^2+^ signaling genes (Table 4).

**Table 4:** Expression levels of genes associated with plant–pathogen interactions in mung bean

Gene_ID	Annotation	Expression level(log2FC)					
		D1 vs. CK1	D4 vs. CK4	D1 + S vs. CK1	D4 + S vs. CK4	D1 + S vs. D1	D4 + S vs. D4
106,758,988	calcium-binding protein CML42	1.58	1.15	2.06	1.36	0.48	-0.19
106,752,538	probable calcium-binding protein CML35	1.10	1.42	1.07	0.74	-0.03	-0.09
106,758,782	probable calcium-binding protein CML45	0.77	1.78	1.04	1.64	0.27	0.20
106,763,935	probable calcium-binding protein CML26	1.31	1.52	1.57	0.70	0.26	0.78
106,764,412	probable calcium-binding protein CML44	3.59	5.27	4.30	0.67	0.71	0.70
106,767,440	probable calcium-binding protein CML41	2.22	2.29	2.45	2.73	0.23	-0.18
106,759,275	probable calcium-binding protein CML27	0.73	1.50	1.00	0.73	0.26	-0.06
106,765,097	calcium-dependent protein kinase 29	1.31	1.80	1.21	1.70	-0.10	0.24
106,768,380	calcium-dependent protein kinase 2	0.64	0.51	1.22	0.01	0.57	-0.30
106,777,786	calcium-dependent protein kinase 28-like	0.80	1.65	0.79	2.30	-0.01	0.88
106,768,224	calcium-dependent protein kinase SK5	-0.49	-0.39	-0.80	-1.21	-0.31	-0.14
106,767,572	calmodulin	0.86	1.57	2.06	1.01	1.20	0.61
106,771,662	calmodulin	0.62	1.21	0.88	1.20	0.26	0.39
106,767,275	calmodulin-like protein 1	1.68	1.83	1.84	0.71	0.15	-0.36
106,778,452	calmodulin-like protein 7	0.47	1.52	1.29	1.67	0.82	-0.32
106,770,817	calmodulin-like protein 11	0.64	1.94	1.91	4.14	1.27	1.74
106,764,918	endoplasmin homolog	0.65	2.05	1.56	1.22	0.91	0.57
106,764,799	heat shock cognate protein 80	1.33	0.90	1.68	-0.24	0.35	-0.04
106,764,653	mitogen-activated protein kinase kinase kinase 1	1.35	0.24	1.12	0.19	-0.23	0.59
106,763,139	pathogenesis-related genes transcriptional activator PTI6-like	2.25	1.97	2.73	0.86	0.47	-0.20
106,775,899	respiratory burst oxidase homolog protein B	2.20	3.28	2.54	3.23	0.34	0.87
106,771,909	somatic embryogenesis receptor kinase 2	1.54	1.40	1.48	0.98	-0.06	-0.56
106,752,427	WRKY transcription factor WRKY24	2.03	3.54	1.72	1.55	-0.32	-0.49

The genes in the significantly enriched plant–pathogen interaction pathway with one log_2_(FC) > 2 in the four treatments are listed

### Differential expression of ribosomal proteins

The ribosome is a large assembly of proteins and ribosomal RNAs (rRNAs) that functions to translate messenger RNAs (mRNAs) into proteins. Ribosome genes were significantly enriched in the D1 + S treatment. The expression levels of DEGs in this pathway are shown in Table S3. The expression levels of all ribosome genes were notably downregulated (log_2_(FC) > 1) after chilling stress with or without UNZ, except for 40S ribosomal protein S3a and 60S ribosomal protein L10-like, which were notably upregulated. The log_2_(FC) values of 23 of 26 genes were positive in D1 + S vs. D1, indicating that UNZ treatment effectively prevented further downregulation of the expression of ribosomal genes, while the log_2_(FC) values of only 11 of 26 genes were positive in D4 + S vs. D4, indicating that UNZ treatment did not effectively prevent ribosomal genes from being further downregulated with the extension of chilling stress.

### Differential expression of cutin, suberin and wax biosynthesis genes

Cutin, suberin and wax, which are involved in cell wall structure, have the principal function of establishing the boundary between the cell and the environment [26]. The expression levels of all differentially expressed cutin, suberin and wax genes were obviously downregulated, except cytochrome P450 86A8, which was upregulated in the 4 treatments, indicating that the synthesis of cutin, suberin and wax was decreased (Table S4). Only peroxygenase 4 in D1 + S vs. D1 and ECERIFERUM 1 in D4 + S vs. D4 were obviously downregulated, indicating that UNZ treatment effectively prevented the expression of these two genes.

### Differential expression of chlorophyll and photosynthesis biosynthesis genes

Chlorophyll is an important photosynthetic pigment in the chloroplasts of plants, and the metabolism of chlorophyll is an important factor in determining crop yield. The contents of Chl a, Chl b, total CHL and Car in leaves were found to increase after UNZ application. To investigate whether chlorophyll synthesis-related genes were involved in the increase in chlorophyll content, we further studied the expression patterns of regulatory enzymes involved in chlorophyll biosynthesis. One significantly enriched pathway among the DEGs was porphyrin and chlorophyll metabolism. The genes involved in this pathway and their expression are listed in Table 5. There are 17 enzymes required for chlorophyll biosynthesis, from glutamyl-tRNA to chlorophyll b [27, 28]. Ten genes were differentially expressed, of which 8 genes were downregulated in the 4 treatments. Staygreen protein and chlorophyll *b* reductase, which are associated with chlorophyll degradation, were found to be upregulated [29, 30]. The log_2_(FC) values of 8 and 9 of these ten genes were positive in D1 + S vs. D1 and D4 + S vs. D4, respectively, indicating that UNZ treatment prevented further downregulation of the expression of chlorophyll biosynthesis genes.

**Table 5:** Expression levels of genes associated with the porphyrin and chlorophyll metabolism synthesis pathway (Vra00860) in mung bean

Gene ID	Annotation	Expression level(log_2_(FC))					
		D1 vs. CK1	D4 vs. CK4	D1 + S vs. CK1	D4 + S vs. CK4	D1 + S vs. D1	D4 + S vs. D4
106,774,182	chlorophyll(ide) b reductase NYC1	1.41	0.90	1.32	1.07	-0.08	0.18
106,761,889	delta-aminolevulinic acid dehydratase	-0.33	-1.10	-0.07	-0.97	0.26	0.13
106,776,114	divinyl chlorophyllide a 8-vinyl-reductase	-0.54	-1.75	-0.01	-1.37	0.53	0.39
106,768,993	geranylgeranyl diphosphate reductase	-0.39	-1.34	-0.10	-1.38	0.29	-0.04
106,762,568	glutamyl-tRNA reductase 1	-0.07	-1.05	-0.11	-0.95	-0.04	0.10
106,764,794	protochlorophyllide reductase	-0.88	-2.06	-0.83	-1.90	0.05	0.16
106,776,119	magnesium-chelatase subunit ChlI	-0.60	-1.21	-0.17	-1.10	0.44	0.11
106,753,941	magnesium-protoporphyrin IX monomethyl ester [oxidative] cyclase	-1.19	-1.31	-0.86	-1.45	0.33	-0.14
106,768,779	magnesium protoporphyrin IX methyltransferase	-0.95	-1.67	-0.34	-1.39	0.60	0.28
106,776,916	STAY-GREEN	-0.16	1.14	-0.03	1.13	0.13	-0.01

The genes in the significantly enriched porphyrin and chlorophyll metabolism synthesis pathway with log_2_(FC) > 2 in at least one of the four treatments are listed

The majority of photosynthetic energy is harnessed via linear electron flow involving light-stimulated electron transfer between two reaction centers, PSI and PSII [31]. According to the results of transcriptome sequencing, 31 genes that were differentially regulated in the 4 treatments were annotated as photosystem genes, and their expression levels are listed in Table 6. Nine genes were detected for PSI eleven genes, including 3 oxygen-evolving enhancer proteins, were detected for PSII; and nine genes, including cytochrome b6-f complex iron-sulfur subunit, plastocyanin and ferredoxin-NADP, ferredoxin reductase, photosynthetic NDH subunit of luminal location and ATP synthase, were detected for photosynthetic electron transfer. The log_2_(FC) values of 15 and 27 of these 31 genes were positive in D1 + S vs. D1 and D4 + S vs. D4, respectively, indicating that UNZ treatment prevented further downregulation of the expression of photosynthetic genes under chilling stress and improved the cold tolerance of mung bean.

**Table 6:** DEGs mapped to the photosynthesis pathway Vra00195

Gene ID	Annotation	Expression level(log_2_(FC))					
		D1 vs. CK1	D4 vs. CK4	D1 + S vs. CK1	D4 + S vs. CK4	D1 + S vs. D1	D4 + S vs. D4
106,754,761	photosystem I subunit O	0.71	-2.73	0.51	-2.70	-0.20	0.03
106,759,560	photosystem I reaction center subunit XI	0.13	-2.58	-0.23	-2.58	-0.36	0.00
106,760,980	photosystem I reaction center subunit psaK	-0.06	-1.77	-0.27	-1.56	-0.21	0.21
106,762,156	photosystem I reaction center subunit VI-2	0.40	-1.49	0.43	-1.23	0.03	0.26
106,762,387	photosystem I reaction center subunit V	-0.25	-1.55	-0.61	-1.68	-0.36	-0.13
106,763,549	photosystem I reaction center subunit N	-0.56	-1.37	-0.71	-1.34	-0.15	0.03
106,765,722	photosystem I reaction center subunit III	0.00	-1.66	0.01	-1.40	0.00	0.26
106,773,542	photosystem I reaction center subunit VI-2	0.25	-1.09	0.24	-0.98	0.00	0.11
106,776,372	photosystem I reaction center subunit IV A	0.47	-1.49	0.45	-1.38	-0.02	0.12
106,757,657	photosystem II reaction center W protein	-0.24	-1.98	-0.31	-1.69	-0.06	0.30
106,770,807	photosystem II reaction center W protein	0.00	-1.48	-0.18	-1.44	-0.18	0.04
106,770,959	photosystem II reaction center PSB28 protein	-0.88	-1.30	-0.20	-1.29	0.68	0.01
106,755,585	photosystem II core complex proteins psbY	-0.68	-1.50	-0.46	-1.18	0.21	0.32
106,767,622	photosystem II core complex proteins psbY	-0.91	-2.21	-0.72	-1.82	0.19	0.39
106,754,237	photosystem II repair protein PSB27-H1	-1.59	-2.38	-0.83	-1.92	0.76	0.46
106,761,826	photosystem II 10 kDa polypeptide	-1.15	-1.51	-1.28	-1.08	-0.12	0.44
106,764,695	oxygen-evolving enhancer protein 2	0.04	-2.70	-0.06	-2.84	-0.10	-0.14
106,768,113	oxygen-evolving enhancer protein 1	0.12	-1.97	0.20	-1.61	0.08	0.36
106,778,062	oxygen-evolving enhancer protein 1	-0.41	-2.00	-0.52	-1.76	-0.10	0.24
106,778,792	oxygen-evolving enhancer protein 2	0.50	-1.78	0.40	-1.68	-0.10	0.10
106,759,740	ATP synthase subunit b	-0.59	-1.51	-0.41	-1.03	0.18	0.48
106,766,849	ATP synthase delta chain	-0.33	-1.78	-0.08	-1.51	0.25	0.28
106,754,175	cytochrome b6-f complex iron-sulfur subunit	0.25	-1.81	0.33	-1.38	0.08	0.43
106,769,577	ferredoxin-1	-0.35	-1.06	-0.10	-0.86	0.25	0.20
106,769,862	ferredoxin	1.02	1.51	0.68	0.88	-0.34	-0.63
106,777,860	ferredoxin	-0.61	-1.54	-0.52	-1.24	0.09	0.31
106,777,632	ferredoxin–NADP reductase	0.08	-1.58	0.02	-1.38	-0.06	0.20
106,758,773	psbP-like protein 1	0.19	-1.77	0.53	-1.63	0.34	0.13
106,771,325	photosynthetic NDH subunit of lumenal location 1	-0.72	-1.64	-0.49	-1.34	0.23	0.30
106,778,556	photosynthetic NDH subunit of lumenal location 3	0.28	-1.38	0.42	-1.09	0.14	0.28
106,759,922	plastocyanin	-0.75	-2.18	-0.48	-1.85	0.27	0.33

The genes in the significantly enriched photosynthesis pathway with log_2_(FC) > 2 in at least one of the four treatments are listed

### Quantitative Real-time PCR verification

**Fig. 4:**
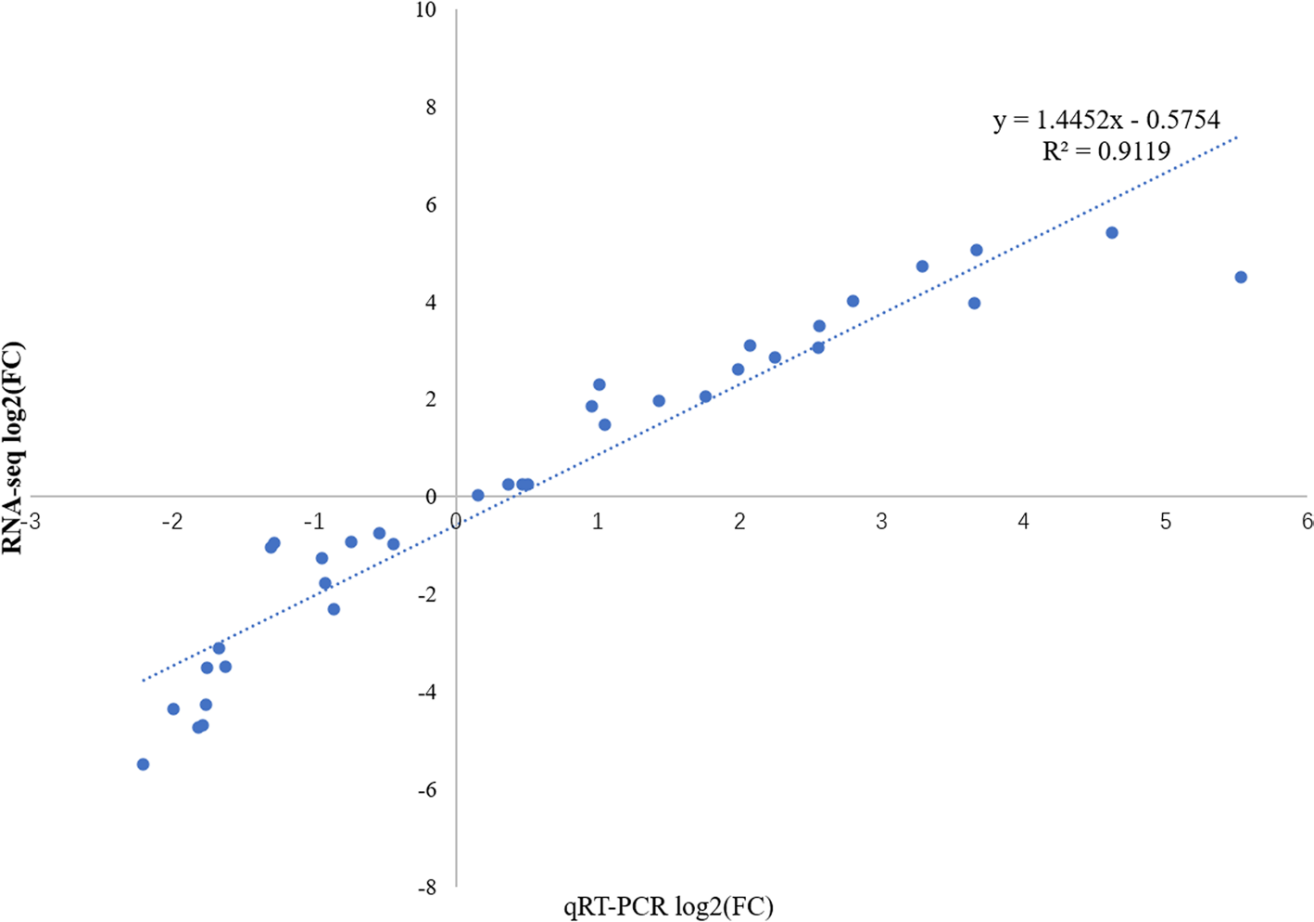
The correlation of RNA-seq and qRT–PCR results

Ten randomly selected cold tolerance unigenes were selected to verify the reliability and accuracy of our transcriptome data using qRT–PCR. The gene IDs and their functional annotations are listed in Table S1. The expression patterns determined by qRT–PCR were compared with those of the RNA-seq assay. The qRT–PCR and RNA-seq results showed a positive correlation coefficient (Pearson coefficient *R*^2^ = 0.911) (Fig. 4).

## Discussion

### Effects of chilling stress and UNZ application on yield and photosynthetic pigments in mung bean

Low-temperature stress can cause changes in membrane phase and membrane permeability in plants, and it can also destroy enzyme systems, cause metabolic disorders, reduce energy supplies, inhibit photosynthesis, promote the accumulation of toxic substances, and affect plant growth and biomass production [32]. Different mung bean varieties differ in their tolerance to cold stress [2, 12]. UNZ has been increasingly applied in crops to increase crop output and quality [13–15, 33]. Two major cultivated varieties, Lvfeng 2 and Lvfeng 5, were selected to evaluate the influence of UNZ application under chilling stress. Our results demonstrated that chilling stress for 1 day and 4 days significantly decreased the yields and chlorophyll content in the two mung bean varieties, although different genotypes of mung bean had different tolerances to chilling stress. Soybean plants under water deficit stress and mung bean plants under waterlogging stress were treated with UNZ, and the chlorophyll content and seed yield were increased compared to those of the control [16, 34]. Our results were consistent with the findings in soybean and mung bean plants. Application of UNZ before chilling stress increased the yields and chlorophyll content significantly in two mung bean varieties compared to the chilling stress treatments. These results indicated that the application of UNZ alleviated chilling stress and that UNZ-induced tolerance to stress was related to changes in photosynthesis and thus increased the yield of mung bean.

### Transcription patterns of chlorophyll and photosynthesis in response to chilling stress and UNZ application

Chlorophyll is an important photosynthetic pigment in the chloroplasts of plants that performs the essential process of harvesting light energy in antenna systems [35]. Chlorophyll metabolism is an important factor in determining the photosynthetic rate and affects crop yield. To date, several studies have focused on the involvement of UNZ in photosynthesis [13, 14, 16, 34], but the expression of chlorophyll synthesis genes after the application of UNZ has been studied only in duckweed [14]. In this study, a genome-wide transcriptomic analysis method was used to investigate the metabolism of key enzymes involved in the chlorophyll biosynthesis pathway in mung bean. Eight of the ten DEGs required for chlorophyll biosynthesis from glutamyl-tRNA to chlorophyll b [27] were dramatically downregulated in the 4 treatments, and two chlorophyll degradation genes were upregulated [28, 29]. The log_2_(FC) of most chlorophyll biosynthesis genes was positive in D1 + S vs. D1 and D4 + S vs. D4, indicating that UNZ treatment prevented further downregulation of the expression of chlorophyll biosynthesis genes by chilling stress. UNZ enhanced the chlorophyll content and upregulated the expression of key enzymes involved in the chlorophyll biosynthesis pathway. These results were consistent with the upregulation of chlorophyll biosynthesis genes after UNZ application [14, 16, 36, 37].

### Transcription patterns of KEGG pathways in response to chilling stress and UNZ application

Mung bean plants often upregulate the expression of some cold-related genes (CORs) to cope with low temperatures. The number of upregulated CORs with a fold change ≥ 3 or downregulated CORs with a fold change ≤ 0.3 genes in mung bean-resistant varieties was 2 times greater than that in susceptible varieties. These CORs are involved in photosynthesis, cellular redox homeostasis, disease resistance and membrane stabilization [11]. Mung bean cell protection and stress-regulated genes were found to respond to low temperatures. More than 489 uniESTs among 1,198 ESTs were involved in metabolic activity, photomorphogenesis, photosynthesis and/or developmental programs [12]. These studies were not conducted at the global transcriptomic level. The use of transcriptomics to quantify the nearly complete set of cellular transcripts allows quantitative and qualitative differences in gene expression to be determined for a specific developmental stage or stress condition [38]. In this study, RNA-seq was used to study the molecular mechanisms underlying UNZ-induced low-temperature tolerance in mung bean. The significantly enriched pathways were cutin, suberin and wax biosynthesis; plant–pathogen interaction; ribosome; porphyrin and chlorophyll metabolism; and photosynthesis.

It is well known that Ca^2+^ acts as a key messenger in regulating cold stress signal transduction pathways [39]. Here, the expression of important Ca^2+^ signaling genes, including reactive oxygen species (ROS), Ca^2+^-dependent protein kinases (CDPKs), mitogen-activated protein kinase (MAPK) cascades and transcription factors (TFs), was found to be upregulated after chilling stress with or without UNZ treatment. The results were consistent with those in other plants exposed to low temperature [15, 40–43]. UNZ treatment before chilling stress upregulated the expression of some Ca^2+^ signal genes and promoted the tolerance of mung bean to chilling stress.

The plant cuticle is the first protective barrier against environmental stress. Most cuticle-associated genes, including those involved in cuticle lipid synthesis, export of cuticular lipids, and regulation of plant cuticle development, are upregulated under low-temperature treatment [44]. In our experiment, most of these genes were downregulated after chilling stress with or without UNZ application, but UNZ treatment effectively prevented further downregulation of the expression of these genes. Our results were obviously different from those showing that most cuticle-associated genes exhibited higher expression levels under cold conditions in *Thellungiella salsuginea* [44]. The reason may be that the plants were treated with different temperatures and at different stages. *Thellungiella salsuginea* was stressed by exposure to 4 ℃ at the preflowering stage, whereas in our experiment, mung beans were stressed by exposure to 15 ℃ at the flowering stage.

## Conclusions

The application of UNZ affected yield and photosynthetic parameters. UNZ effectively delayed the decrease in the photosynthetic pigment content of leaves under chilling stress and accelerated the increase in photosynthetic pigment content in leaves during the recovery period, thus effectively alleviating the decline in mung bean yield. Genes in four pathways (cutin, suberin and wax biosynthesis; photosynthesis; porphyrin and chlorophyll metabolism; and ribosome) were downregulated, while genes related to plant–pathogen interaction were upregulated, by chilling stress. UNZ treatment effectively altered the expression patterns of these genes and increased mung bean tolerance to chilling stress.

## Methods

### Plant materials and UNZ treatments

Two cultivars of mung bean, Lvfeng 2 and Lvfeng 5, were provided by the germplasm bank of the National Coarse Cereals Engineering Technology Research Center for use as materials. The two cultivars were planted in pots and grown to the outset of the flowering stage (R1) in a greenhouse at Heilongjiang Academy of Agricultural Sciences in 2017. Three treatments were established, and each treatment was repeated 4 times. The plants were sprayed with water and maintained at the natural temperature for 4 days (CK); then, the plants were sprayed with water, and 36 h later, the plants were shifted to 15 ℃ for 1 d, 2 d, 3 d or 4 d (D1, D2, D3, D4) and then returned to the warmer temperature to grow for 4 days; the plants were sprayed with 50 mg·L^−1^ UNZ (Jiangxi Nongda Ruite Chemical Technology Co., Ltd., CAS: 76714–83-5), and 36 h later, the plants were shifted to 15 ℃ to grow for 1 d, 2 d, 3 d and 4 d (D1 + S, D2 + S, D3 + S, D4 + S) and then returned to the warmer temperature to grow for 4 d [6, 7]. The plants of the 3 treatments were grown under natural conditions until maturity. The lowest external temperature of each day during the 4 days of chilling stress was above 20 °C, as monitored by an EM50 micro meteorological monitoring system.

### Yield components and chlorophyll measurement

After mung bean was grown to maturity, the pod number per plant, particle number per plant, and yield were measured at the maturity stage (R8). The mung bean grain was dried to constant weight, and the final yield was calculated according to the determined 14% water content. Each treatment consisted of 4 replicates with 10 plants per replicate. The mung bean leaves at each day of chilling stress and recovery were taken as the materials to determine the chlorophyll content. Fresh leaves (0.1 g) were immersed in 10 mL alcohol and kept at room temperature for 24 h and shielded from light for 1 d after chilling stress. The leaf chlorophyll a, chlorophyll b and carotenoid concentrations in the supernatant of the solution were measured using a spectrophotometer at 663 and 645 nm and 470 nm, respectively [45, 46].

### RNA extraction, cDNA library construction and sequencing

Lvfeng 2 was used as the experimental material. There were 6 treatments (CK1, D1 and D1 + S and CK4, D4 and D4 + S), which are described in the plant materials and UNZ treatments section. Each treatment consisted of 3 replicates. Sample collection was carried out between 8:00 and 9:00 a.m. Mixed samples of three leaves each in the CK, D and D + S treatments were subjected to RNA extraction (Invitrogen TRIzol Reagent of RNA extraction kit 15,596,018). A total of eighteen libraries were constructed using a NEBNext Ultra RNA Library Prep Kit for Illumina (NEB, USA) and sequenced using an Illumina HiSeq^TM^2000 (Beijing Biomarker Technologies Co.).

### Assembly and functional annotation

The raw sequencing reads were cleaned by removing adaptors and low-quality reads. After filtering, Bowtie software (version 2.2.5) was used to map the clean reads to the mung bean (VC1973A) reference genome (463.638 Mbp) [47], and then RSEM [48] was used to estimate the expression levels with the FPKM value. To identify DEGs, the criteria applied were an FDR (false discovery rate) less than 0.01 and an absolute value of log2 ratio of at least 1. The screened DEGs were analyzed mainly by GO function (http://geneontology.org/system [49] and KEGG pathway enrichment (https://www.kegg.jp/kegg/kegg1.html) [25]. GO annotations of DEGs and GO functions mapped to the corresponding secondary features based on unigene GO annotation were extracted, and a histogram was drawn [49]. The KEGG pathway enrichment analysis was implemented via KOBAS2.0 (http://kobas.cbi.pku.edu.cn/home.do) [25].

### Quantitative real-time PCR verification

Quantitative real-time PCR (qRT–PCR) was performed to examine the expression patterns shown by the RNA-seq analysis. Ten genes were selected on the basis of their potential functions in RNA-seq. The sequences of each primer are shown in Table S1. RNA (1 μg) from each treatment was treated with DNAse I (Invitrogen), translated into first-strand cDNA with TransScript One-Step gDNA Removal (Super Script) and cDNA Synthesis SuperMix (Transgene Biotech), and then the cDNA was stored at − 20 °C for subsequent analysis. Each PCR contained a 20 μl mixture consisting of 2 μl of cDNA, 10 μl of 2 × TransStart Top Green qPCR SuperMix, and 0.4 μl of the forward and reverse primers. All qRT-PCRs were performed in three technical replicates in a Bio–Rad CFX96 thermocycler and performed in two steps: predenaturation for 2 min at 94 °C, followed by 45 cycles of denaturation for 2 s at 94 °C and annealing/extension for 15 s at 60 °C. The relative expression level was calculated by the 2^−ΔΔCt^ method [50] with the actin gene as an internal standard. Each measurement included three biological and three technical replicates.

## Supplementary Information

**Additional file 1: Table S1.** Selected genes and primers forquantitative qRT–PCR.

**Additional file 2: Table S2.** Effect of uniconazole on DEGs in mungbean leaves under chilling stress at the Rl stage.

**Additional file 3: Table S3.** Expression level of genes associatedwith ribosome (Vra03010) in mung bean.

**Additional file 4: Table S4.** Expression level of genes associatedwith cutin, suberin and wax biosynthesis(Vra00073) in mung bean.

**Additional file 5: Figure S1.** Effects of uniconazole on photosynthetic pigments in Lvfeng 5 under chillingstress at the R1 stage.

**Additional file 6: Figure S2.** Pearsoncorrelation between samples.

## Data Availability

The datasets used and/or analyzed during the current study are available from Mendeley Data, v1 (http://dx.doi.org/10.17632/v9b8pcxk56.1).

## Abbreviations

UNZ: Uniconazole
DEGs: Differentially expressed genes
Chl a: Chlorophyll a
Chl b: Chlorophyll b
Chl (a + b): Total chlorophyll
Car: Carotenoids
