# Supplementary material for: Transcriptomic analysis of Vigna radiata in response to chilling stress and uniconazole application
Source: BMC Genomics. 2022 Mar 14;23:205. doi: 10.1186/s12864-022-08443-6 (PMC8922894; doi:10.1186/s12864-022-08443-6)
Supplement: Supplementary file 1 — Additional file 1: Table S1. Selected genes and primers forquantitative qRT–PCR. [file 12864_2022_8443_MOESM1_ESM.docx]

Table S1 Selected genes and primers for quantitative qRT–PCR

| Gene | GeneID | Annotation | Primers |
| --- | --- | --- | --- |
| PsbO | LOC106778062 | oxygen-evolving enhancer protein 1 | 5'-GCGAGGTTATCGGTGTGTTC-3'  5'-GATAATGCAGCAACAGCATG-3' |
| PsbP | LOC106778792 | oxygen-evolving enhancer protein 2 | 5'-GCAGCAACTGTGAAAGATGG-3'  5'-ATTCAACACATACACACCCC-3' |
| PsbQ | LOC106758773 | psbP-like protein 1 | 5'-GAAGCTTCAAGGAAGAAAAG-3'  5'-GGAATCAGCATGGCATACCAA-3' |
| PsbY | LOC106767622 | photosystem II core complex proteins psbY | 5'-GACTATTGCTTTCCAATTAAC-3'  5'-AGAATTAAGAATACCCACGAG-3' |
| Psb28 | LOC106770959 | photosystem II reaction center PSB28 protein | 5'-AGGAGTCCTTCAGTCGGTTG-3’  5'-CAGAGTTAGCACATTGACTC-3’ |
| SUS | LOC106775028 | sucrose synthase 6 | 5'-CGAGTCTCAAATCGCGTAGC-3'  5'-CAGAGCTTCTCCGTTTGATG-3’ |
|  | LOC106762931 | sucrose synthase 7-like | 5'-TTATACCTTCTGGAGGCGGG-3’  5'-TGCAATCCAGACTGTGAACG-3’ |
| POD | LOC106776936 | peroxidase 51 | 5'-TGACTGGCCTCTTCTTTGAG-3’  5'-GAGACCATAGGTTTAGAATTC-3’ |
| SOD | LOC106770881 | superoxide dismutase | 5'-CTGCACTGATGCTTCTCAGG-3’  5'-AGTGGGGTGGCTACAGATGC-3 |
| Actin | LOC10675756) | actin-3 | 5'-GCACCACCAGAGAGGAAATAC-3'  5'-TCATACTCAGCCTTCGCAATC-3' |
|  |  |  |  |
