# Supplementary material for: Transcriptomic analysis of Vigna radiata in response to chilling stress and uniconazole application
Source: BMC Genomics. 2022 Mar 14;23:205. doi: 10.1186/s12864-022-08443-6 (PMC8922894; doi:10.1186/s12864-022-08443-6)
Supplement: Supplementary file 2 — Additional file 2: Table S2. Effect of uniconazole on DEGs in mungbean leaves under chilling stress at the Rl stage. [file 12864_2022_8443_MOESM2_ESM.docx]

Table S2 Effect of uniconazole on DEGs in mung bean leaves under chilling stress at the Rl stage

| Combination | Upregulation | Downregulation | All DEGs |
| --- | --- | --- | --- |
| D1 vs. CK1 | 2 023 | 2 002 | 4 025 |
| D1+S vs. CKl | 2 449 | 2 456 | 4 905 |
| D4 vs. CK4 | 2 199 | 1 824 | 4 023 |
| D4+S vs. CK4 | 1 752 | 1 514 | 3 266 |
