# Supplementary material for: Transcriptomic analysis of Vigna radiata in response to chilling stress and uniconazole application
Source: BMC Genomics. 2022 Mar 14;23:205. doi: 10.1186/s12864-022-08443-6 (PMC8922894; doi:10.1186/s12864-022-08443-6)
Supplement: Supplementary file 3 — Additional file 3: Table S3. Expression level of genes associatedwith ribosome (Vra03010) in mung bean. [file 12864_2022_8443_MOESM3_ESM.docx]

Table S3 Expression level of genes associated with ribosome (Vra03010) in mung bean

| Gene ID | Annotation | Expression level（log_2_(FC)） | | | | | |
| --- | --- | --- | --- | --- | --- | --- | --- |
|  |  | D1 vs.  CK1 | D4 vs.  CK4 | D1+S  vs. CK1 | D4+S  vs. CK4 | D1+S  vs. D1 | D4+S  vs. D4 |
| 106759626 | 50S ribosomal protein L5 | -0.45 | -1.60 | -0.14 | -1.62 | 0.32 | -0.01 |
| 106772956 | 40S ribosomal protein SA-like | -0.41 | -1.12 | -0.09 | -1.33 | 0.33 | -0.22 |
| 106768186 | 30S ribosomal protein S1 | 0.17 | -1.66 | 0.02 | -1.55 | -0.15 | 0.11 |
| 106767138 | 40S ribosomal protein S15a-like | -0.61 | -1.28 | 0.05 | -1.52 | 0.65 | -0.24 |
| 106753293 | 60S ribosomal protein L27a-3 | -0.41 | -1.04 | 0.00 | -1.13 | 0.41 | -0.10 |
| 106760829 | 40S ribosomal protein SA | 0.01 | -1.04 | 0.33 | -1.36 | 0.32 | -0.31 |
| 106775262 | 40S ribosomal protein S7 | -0.27 | -0.90 | 0.05 | -1.09 | 0.32 | -0.19 |
| 106768630 | 40S ribosomal protein S13 | -0.24 | -0.98 | 0.12 | -1.14 | 0.36 | -0.17 |
| 106756633 | 50S ribosomal protein L13 | -0.76 | -1.71 | -0.24 | -1.49 | 0.52 | 0.22 |
| 106775970 | 60S ribosomal protein L10-like | 0.21 | 1.18 | 0.26 | 1.20 | 0.05 | 0.02 |
| 106775059 | 50S ribosomal protein L17 | -0.81 | -1.38 | -0.20 | -1.23 | 0.61 | 0.15 |
| 106774979 | 50S ribosomal protein L6 | -0.27 | -1.24 | 0.05 | -1.15 | 0.32 | 0.08 |
| 106761560 | 40S ribosomal protein S3a | 1.45 | 2.60 | 1.25 | 2.39 | -0.20 | -0.21 |
| 106754691 | 60S ribosomal protein L9-like | -0.31 | -0.90 | 0.03 | -1.18 | 0.33 | -0.28 |
| 106774353 | 50S ribosomal protein L4 | -0.48 | -1.49 | -0.18 | -1.23 | 0.30 | 0.26 |
| 106770917 | 60S ribosomal protein L23 | -0.16 | -1.08 | 0.31 | -1.23 | 0.48 | -0.15 |
| 106754183 | 40S ribosomal protein S5 | -0.48 | -0.82 | 0.04 | -1.05 | 0.52 | -0.23 |
| 106776383 | 40S ribosomal protein S3a | -0.12 | -0.82 | 0.40 | -1.02 | 0.51 | -0.20 |
| 106776014 | 50S ribosomal protein L4 | -0.64 | -1.09 | -0.41 | -1.03 | 0.23 | 0.05 |
| 106764118 | 50S ribosomal protein L31 | -0.56 | -1.24 | -0.37 | -1.03 | 0.19 | 0.21 |
| 106763543 | 60S ribosomal protein L9 | -0.11 | -0.90 | 0.26 | -1.08 | 0.37 | -0.18 |
| 106780207 | 60S ribosomal protein L11 | -0.29 | -0.95 | 0.07 | -1.19 | 0.36 | -0.24 |
| 106775690 | 50S ribosomal protein L11 | 0.02 | -1.08 | 0.22 | -0.94 | 0.19 | 0.14 |
| 106771030 | 60S ribosomal protein L22-2 | -0.30 | -0.78 | 0.03 | -1.06 | 0.33 | -0.28 |
| 106767033 | 30S ribosomal protein S6 alpha | -0.58 | -1.05 | -0.09 | -0.98 | 0.49 | 0.07 |
| 106759824 | 50S ribosomal protein L27 | 0.14 | -1.19 | 0.08 | -1.00 | -0.06 | 0.18 |

The genes in significantly enriched ribosome pathway with log_2_(FC) >2 in the four treatments are listed.
