# Supplementary material for: Transcriptomic analysis of Vigna radiata in response to chilling stress and uniconazole application
Source: BMC Genomics. 2022 Mar 14;23:205. doi: 10.1186/s12864-022-08443-6 (PMC8922894; doi:10.1186/s12864-022-08443-6)
Supplement: Supplementary file 4 — Additional file 4: Table S4. Expression level of genes associatedwith cutin, suberin and wax biosynthesis(Vra00073) in mung bean. [file 12864_2022_8443_MOESM4_ESM.docx]

Table S4 Expression level of genes associated with cutin, suberin and wax biosynthesis (Vra00073) in mung bean

| Gene ID | Annotation | Expression level（log_2_ (FC)） | | | | | |
| --- | --- | --- | --- | --- | --- | --- | --- |
|  |  | D1 vs.  CK1 | D4 vs.CK4 | D1+S  vs. CK1 | D4+S  vs. CK4 | D1+S  vs. D1 | D4+S  vs. D4 |
| 106755648 | fatty acyl-CoA reductase 3 | -2.82 | -3.35 | -2.09 | 0.01 | 0.73 | 0.04 |
| 106759419 | fatty acyl-CoA reductase 3 | -1.61 | -2.16 | -1.39 | -1.48 | 0.23 | -0.88 |
| 106758926 | fatty acyl-CoA reductase 3 | -2.94 | -1.98 | -2.74 | 1.16 | 0.20 | -0.42 |
| 106772969 | protein ECERIFERUM 1 | -7.59 | 0.25 | -7.06 | 0.34 | 0.53 | -4.96 |
| 106764310 | cytochrome P450 86A1 | -2.62 | -2.22 | -2.84 | 0.17 | -0.22 | -0.38 |
| 106775311 | cytochrome P450 86A1 | -2.90 | -1.87 | -3.51 | -0.23 | -0.61 | -0.91 |
| 106774045 | cytochrome P450 86A8 | 0.40 | 0.68 | 1.37 | 2.00 | 0.98 | -0.49 |
| 106769560 | cytochrome P450 86A22 | -3.29 | -4.88 | -2.37 | -0.43 | 0.91 | 0.37 |
| 106755878 | cytochrome P450 86B1 | -2.60 | -1.25 | -2.76 | 1.08 | -0.17 | -0.36 |
| 106758319 | ω-hydroxypalmitate O-feruloyl transferase | -4.05 | 0.02 | -3.84 | 2.64 | 0.21 | -0.31 |
| 106761500 | ω-hydroxypalmitate O-feruloyl transferase | -2.52 | -2.53 | -3.83 | -1.32 | -1.32 | 0.31 |
| 106777294 | probable peroxygenase 4 | -3.56 | -2.75 | -9.05 | -2.01 | -5.49 | -0.36 |

The genes in significantly enriched cutin, suberin and wax biosynthesis pathway with log_2_(FC) >2 in the four treatments are listed.
