# Supplementary material for: Transcriptomic analysis of Vigna radiata in response to chilling stress and uniconazole application
Source: BMC Genomics. 2022 Mar 14;23:205. doi: 10.1186/s12864-022-08443-6 (PMC8922894; doi:10.1186/s12864-022-08443-6)
Supplement: Supplementary file 5 — Additional file 5: Figure S1. Effects of uniconazole on photosynthetic pigments in Lvfeng 5 under chillingstress at the R1 stage. [file 12864_2022_8443_MOESM5_ESM.docx]

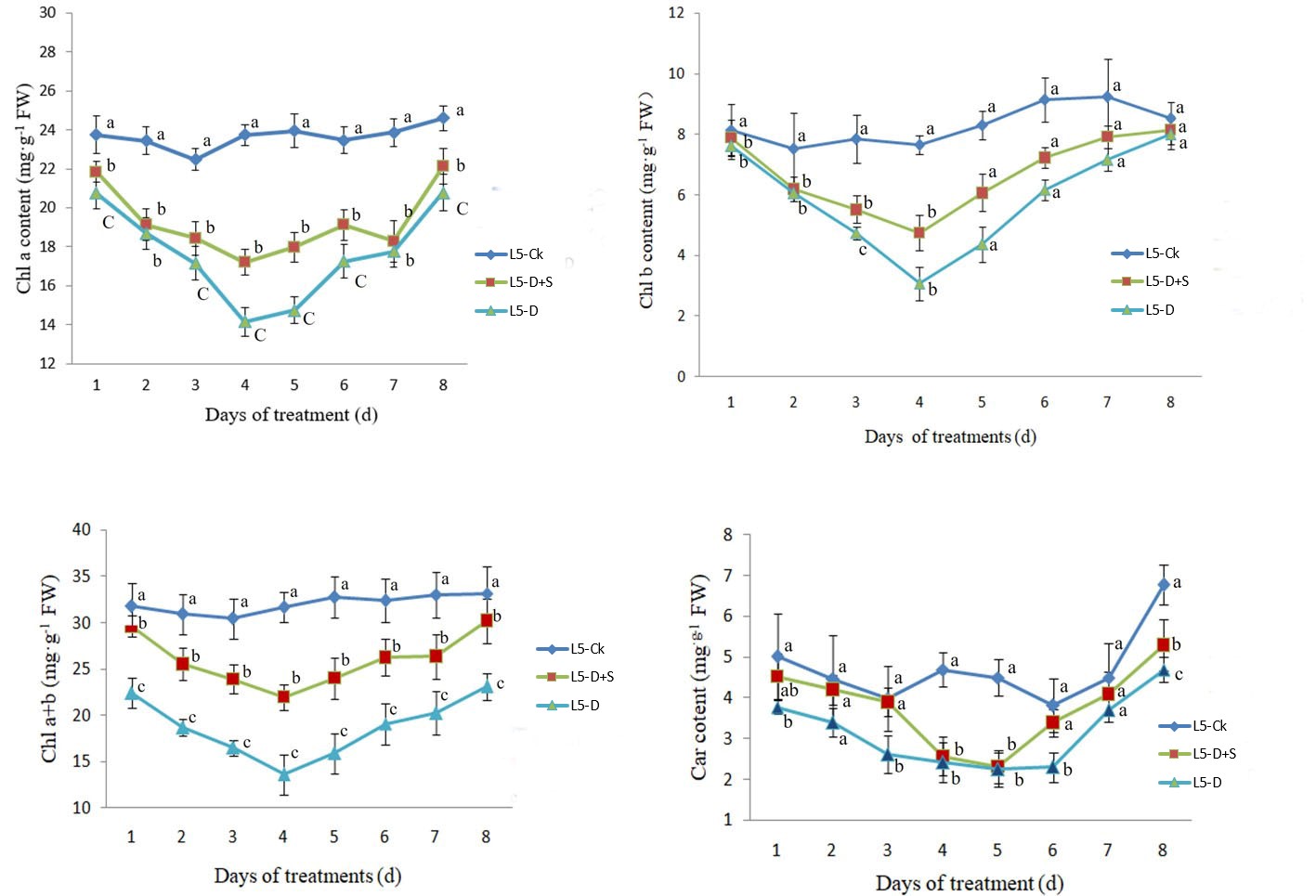


Figure S1 Effects of uniconazole on photosynthetic pigments in Lvfeng 5 under chilling stress at the R1 stage

Note: Data in the figure are the mean ± SD of four replicates. L5-CK, plants grown in the natural environment; L5-D, plants grown in the chilling environment; L5-D+S, plants grown in the chilling environment + 50 mg·L^-1^ UNZ; 1-4, time for chilling stress; 5-8: time for recovery in the natural environment. Different lowercase letters represent significant differences (p<0.05) between the treatment and control.
