# Supplementary figures and images for: Transcriptomic analysis of Vigna radiata in response to chilling stress and uniconazole application
Source: BMC Genomics. 2022 Mar 14;23:205. doi: 10.1186/s12864-022-08443-6 (PMC8922894; doi:10.1186/s12864-022-08443-6)

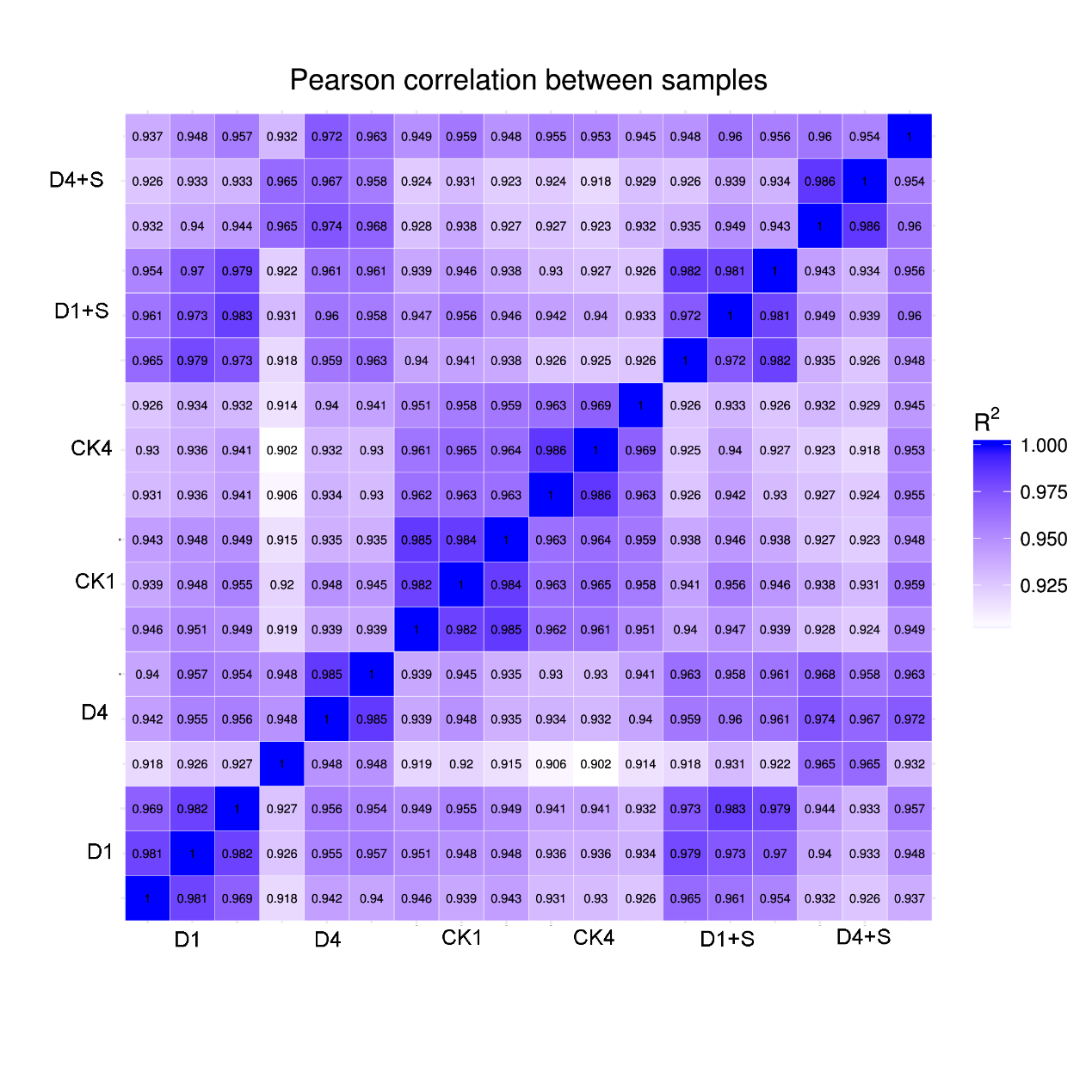


Figure S2 Pearson correlation between samples

Supplement: Supplementary file 6 — Additional file 6: Figure S2. Pearsoncorrelation between samples. [file 12864_2022_8443_MOESM6_ESM.docx]
